# Teaching persuasive essay writing online to first-year undergraduates: A phenomenological study of instructional design and learning experiences

**DOI:** 10.1371/journal.pone.0353171

**Published:** 2026-07-17

**Authors:** Abdul Karim, Md Abdullah Al Mamun, Shahin Sultana, Muhammad Kamarul Kabilan

**Affiliations:** 1 BRAC Institute of Languages, BRAC University, Dhaka, Bangladesh; 2 Universal College Bangladesh, Dhaka, Bangladesh; 3 Faculty of Arts and Humanities, Notre Dame University Bangladesh, Dhaka, Bangladesh; 4 School of Educational Studies, Universiti Sains Malaysia, Penang, Malaysia; 5 Faculty of Letters, Universitas Negeri Malang, East Jawa, Indonesia; 6 Faculty of Teacher Training and Education, Universitas Siliwangi, West Jawa, Indonesia; Bahir Dar University, ETHIOPIA

## Abstract

The teaching and learning of academic essays faced difficulties in in-person classes. Challenges in academic essay instruction were exacerbated in online learning environments during COVID-19. Yet some institutions arranged sophisticated platforms to share instructional materials for imparting English language education online. The focal university was one such progressive institution. Like other courses, English language education was delivered through ‘Arwa’, an online platform developed by the university to support teaching and learning during the pandemic. The phenomenological study aimed to understand how instructional materials designed for an English foundation course contributed to first-year undergraduates’ experiences of learning persuasive essay writing. Data were collected primarily through semi-structured interviews and supplemented by an open-ended questionnaire administered to the same eight participants. Phenomenological thematic analysis was applied to interpret the data. The findings reveal how participants experienced the instructional design and materials as supporting their engagement with outlining, drafting, editing, and revising persuasive essays. Participants also described collaborative writing and rubric-guided peer feedback as meaningful learning experiences that supported their engagement with persuasive essay writing. From a phenomenological perspective, the study contributes to understanding how learners make sense of persuasive essay writing through structured online instruction, collaborative writing, and rubric-guided peer feedback. Pedagogically, the findings highlight the value of sequenced instructional materials, collaborative writing, and guided peer interaction in supporting students’ engagement with persuasive writing in online and blended learning environments.

## Introduction

Globally, developing mastery of academic writing remains a challenge for learners, particularly those studying in English as a Second (ESL) and English as a Foreign Language (EFL) contexts, e.g., China, Japan, Malaysia, Korea, and Vietnam, because the nature of academic writing is quite different from general writing in terms of its purpose, potential readers, text structure, and language style [1–9]. Similar problems have been prevalent in students’ writing in Bangladesh. Studies report that students’ writing difficulties in Bangladesh stem from multiple interconnected factors, including inadequate writing instruction, linguistic limitations, weak foundations in academic writing practices, and affective challenges [10–17]. Institutional factors such as insufficient teacher professional development, examination-oriented teaching, and limited English proficiency among teachers may further constrain students’ opportunities to develop academic writing competence [13,18–24]. Consequently, students often enroll in university without the expected level of academic writing proficiency [25,26].

The conundrums intensified during the height of COVID-19, when educational institutions shifted to online teaching and learning (OTL) [27]. Teachers, ranging from lecturers to senior professors, and students in many countries shifted to online teaching and learning (OTL), whereby teachers had to carefully prepare lectures and materials for the students to digest and absorb the learning fully [28–32]. However, in Bangladesh, OTL was accompanied by several infrastructural and pedagogical challenges, including limited technological access, insufficient digital literacy, and reduced opportunities for guided writing practice, interaction and feedback [33–36]. Similar challenges in comparable ESL/EFL contexts suggest that inadequately designed online instruction may constrain students’ writing development [37]. To elaborate, inappropriately presented learning materials, insufficient time allocated to mentor students, and limited opportunities to provide feedback create resistance, which impairs students’ success in mastering this productive skill [37]. Challenged by the said predicaments, while many educational institutions constantly struggled to continue teaching and learning to disseminate knowledge, some institutions survived the challenges and continued OTL activities with strategic and well-guided initiatives. Among the institutions that took workable measures to minimize the challenges reported above, one of the institutions remains at the focal point of this study.

The OTL at Kiyan University (pseudonym and the research site), which adopted English Medium Instruction (EMI), endured for over two years, equaling half of the graduation period. Thus, skipping the teaching and learning of academic essays was not affordable or logical, provided that in English medium universities, academic writing becomes inevitable as students must write in the examination, complete assignments, prepare lab reports, and accomplish all types of academic activities in English [38,39]. Eventually, courses concerning English for Academic Purposes (EAP) were offered to first-year undergraduates to facilitate the successful accomplishment of academic activities, including academic writing [40,41]. Hence, teaching and learning of academic essays gained notable attention at Kiyan University (the focal university) and became a focus within the university’s online instructional response during the pandemic. Although several studies have examined online writing instructions during COVID-19, their focus has largely lain on isolated tools and feedback practices. For example, research has presented digital writing tools [37], discussed multimedia interventions such as images, videos, and comic-strip creation [42–44] and studied the role of teacher feedback in online platforms [45]. While these studies provide useful insights, they do not comprehensively examine how instructional design, structured materials, collaborative writing, and rubric-driven peer feedback jointly contribute to students’ development of persuasive academic essays in EMI contexts.

Despite growing research on online writing instruction, existing studies provide a limited understanding of how systematically designed online instruction may support persuasive essay writing in EMI contexts through the combined role of structured instructional materials, collaborative writing, and rubric-guided peer feedback. Scholars [e.g., 31,46,47] have called for further investigations into how tertiary-level students are engaged in learning academic writing processes. In addition, Akbana et al. [48], in a review of emergency remote teaching in English language teaching, reported mixed outcomes and highlighted the need for further inquiry into online instructional practices. Addressing this gap may provide insights into how instructional design, collaborative writing, and peer feedback can support academic writing across online, blended, and face-to-face learning environments.

Considering the numerous challenges associated with OTL and ESL/EFL writing [49], the present phenomenological study explores how instructional design and materials (see ‘Methods’ section details) devised for an English foundation course at the focal university may have contributed to first-year undergraduates’ learning of persuasive essay writing. Specifically, the study examines students’ lived experiences of structured online instruction, collaborative writing, and rubric-guided peer feedback in developing persuasive essays.

The study is theoretically informed by Cognitive Load Theory, which explains how intrinsic, extraneous, and germane cognitive load shape learning through the activation of schemata in working memory. In addition, collaborative writing and rubric-driven peer feedback are conceptualized to interpret how structured interaction and guided evaluation support persuasive essay development in online settings. Together, these frameworks provide an analytical lens for examining how instructional sequencing, collaborative engagement, and feedback mechanisms contributed to students’ learning in the OTL platform ‘Arwa’.

### Cognitive Load Theory (CLT)

Cognitive load refers to the total memory resources required to accomplish a learning task [50]. Cognitive Load Theory (CLT) explains learning in relation to the interaction between working memory and long-term memory [51]. Working memory has a limited capacity for processing novel information; consequently, unfamiliar or poorly organized learning materials may impose substantial cognitive demands. Long-term memory, by contrast, stores knowledge in the form of cognitive schemata, which support the organization and retrieval of information and may reduce the burden on working memory during complex learning tasks [52]. When learners process information that is connected to prior knowledge and organized within existing or developing schemata, cognitive processing becomes more efficient [52,53]. Schema automation occurs when learners repeatedly connect previously stored knowledge with new information, enabling them to engage with increasingly complex tasks with reduced pressure on working memory [52].

Building on this foundation, Van Merrienboer and Ayres identified three interrelated dimensions of cognitive load, namely Intrinsic Cognitive Load, Extraneous Cognitive Load, and Germane Cognitive Load. Intrinsic Cognitive Load is “determined by the interaction between the nature of the materials being learned and the level of expertise of the learner” [52]. It depends on the number of elements that must be processed simultaneously in working memory and on the degree of element interactivity within the material. Materials requiring high element interactivity are therefore more complex and demanding for working memory, whereas materials with lower interactivity are comparatively easier to process.

In the present study, persuasive essay writing may involve high element interactivity because learners need to simultaneously process components such as thesis statements, topic sentences, supporting evidence, and coherence. Such demands may become more challenging when learners possess limited prior schemata related to these elements. Familiarity with foundational components, including paragraph structure and topic sentences, may facilitate engagement with more complex writing tasks; thus, instruction that progresses from foundational components to complete essay construction may be interpreted as an approach to managing intrinsic cognitive load.

Extraneous Cognitive Load refers to the load imposed on working memory due to the manner in which information or tasks are presented, rather than the inherent complexity of the task itself [54]. Such a load may arise when instructional materials present information in a scattered or poorly organized manner, requiring learners to search for relevant elements while attempting to complete a task [52]. Consequently, extraneous cognitive load is often associated with instructional inefficiencies. Reducing such load requires instructional materials to be presented coherently and systematically so that learners can direct cognitive resources toward learning tasks rather than toward locating or organizing information.

Germane cognitive load concerns the cognitive effort invested in schema construction and refinement. Repeated engagement with related learning activities may support learners in connecting prior knowledge with increasingly complex writing tasks [52]. For example, students may initially become familiar with components such as topic sentences and supporting details during paragraph writing before engaging with broader essay structures. Such progression may contribute to the refinement and application of existing schemata throughout the learning process.

### The implication of CLT in OTL

A common practice in OTL platforms is the incorporation of complex learning tasks into instructional design. Such tasks often embody multiple interacting elements, requiring students to coordinate various components simultaneously in working memory. Likewise, tasks designed to develop skills require learners to integrate information in working memory to produce coherent performance. From a Cognitive Load Theory perspective, the issue is not solely task complexity but whether multiple interacting elements exceed the limited capacity of working memory, particularly when learners have not yet developed the relevant schemata.

Van Merrienboer and Ayres [52] indicated that the removal of extraneous cognitive load alone does not guarantee effective learning, as materials characterized by high element interactivity may still hinder learning performance. Therefore, they recommend avoiding the simultaneous presentation of all relevant information. Instruction may begin with an initial orientation to the topic or task, allowing learners to become familiar with essential components before engaging in more demanding activities. In this approach, a simpler version of the task is introduced first, followed by progressively more complex versions. This progressive method primarily influences intrinsic cognitive load by reducing element interactivity during the early stages of instruction. As learners gradually develop familiarity with foundational elements, they become better equipped to manage more complex tasks.

In the present study, CLT provides an explanatory lens for interpreting how the staged presentation of instructional materials, collaborative writing activities, and rubric-guided peer feedback may shape learners’ reported experiences of persuasive essay writing. The gradual movement from foundational components to complete essay construction is relevant to the research question because it helps explain how participants experienced the instructional design and materials as supporting their understanding of persuasive essay structure and writing processes. This perspective also informs the interpretation of the findings, particularly participants’ reported movement from fragmented prior understandings toward a more organized understanding of outlining, drafting, revising, and peer feedback.

### Collaborative writing and peer feedback

The increasing use of digital technologies in higher education has expanded opportunities for teaching and learning through digital platforms such as forums, chats, blogs, wikis, Google Docs, and social media [55]. These developments have also increased opportunities for collaborative writing, which contributes to learners’ development of writing proficiency [56,57]. Engaging in collaborative activities gives students the opportunity to address difficulties, as long as the group members strive to help each other using their knowledge and expertise [40]. In collaborative writing, joint writing tasks that require a high level of cognitive competence can be accomplished effectively [58]. Collaborative writing ensures joint participation in writing composition, encouraging learners to deliberately generate ideas and find the best ways to present the ideas [59]. Thus, the fundamental idea of collaborative learning concerns the active involvement of two or more students in contributing to the attainment of a shared learning goal, deploying the effort required to reach this goal, whether face-to-face, computer-mediated, or in blended environments [50,55]. A significant aspect of collaborative writing is students’ engagement in peer feedback, through which learners exchange suggestions, negotiate revisions, and evaluate writing in relation to shared criteria [59–61].

In the process of collaboration, the learners provide each other with “suggestions and counter-suggestions, positive (confirming) and corrective feedback” [59]. Valero Haro et al. [62] reported that peer feedback improved the quality of students’ argumentative essays. Similar findings were reported by Noroozi et al. [61], while Gao et al. [63], in a systematic review, identified peer feedback as an important mechanism through which learners revise, refine, and strengthen their writing.

However, feedback may be ineffective depending on the learning contexts, circumstances, and time frames [55]. López-Pellisa et al. [55] argued that the effectiveness of peer feedback may depend on factors such as the feedback provider, timing, and type of feedback exchanged. Similarly, students in Huisman et al.’s [64] study did not always perceive peer feedback as contributing to improved writing performance. Earlier studies also highlighted learners’ limited ability to evaluate peers’ writing effectively [65,66]. Consistent with these concerns, Banihashem et al. [67] observed that “furnishing high-quality feedback to peers remains a challenge” (p. 2).

Geitz et al. [68] argued that students often participate actively in peer-feedback activities because they perceive their peers as collaborators of equal status rather than as experts. Active participation alone may not ensure meaningful feedback, as learners may lack the evaluative knowledge required to identify weaknesses, justify suggestions, or provide revision-oriented comments. Zhao [69] emphasized the role of teacher support in developing learners’ capacity to evaluate peers’ work and provide constructive feedback. Wang [70] highlighted the value of rubric-guided peer feedback, showing that explicit criteria help learners recognize key dimensions of writing and attend to both language-related and content-related issues. Latifi et al. [71] found that learners who receive guidance on how to provide feedback tend to generate more meaningful comments because the criteria enable them to evaluate arguments and reasoning more systematically. Kerman et al. [72] further noted that peer feedback becomes more useful when it helps learners identify specific problems in a text, as feedback that draws attention to such issues creates opportunities for revision and improvement. Studies by Valero Haro et al. [62,73], and [61] indicated that guided or directed peer feedback is more conducive to writing development than unguided feedback. Er et al. [74] extended this discussion by arguing that peer-feedback activities can foster a dialogic environment in which feedback providers and recipients exchange perspectives, discuss writing-related concerns, and reflect on strengths and weaknesses in their work. In their collaborative learning approach to dialogic peer feedback, Er et al. [74] proposed the use of peer-assessment and self-assessment rubrics to support more focused feedback exchanges and reflective engagement with writing. The literature suggests that the value of peer feedback is shaped not only by learners’ participation but also by the extent to which feedback activities are structured through guidance, explicit evaluation criteria, opportunities for dialogue, and reflection on writing.

### The implication of CLT concerning collaborative writing and peer feedback

The incorporation of collaborative writing activities has implications for both intrinsic and extraneous cognitive load. Persuasive essay writing requires learners to engage simultaneously with multiple interacting elements, including grabbers, connecting ideas, thesis statements, supporting evidence, and overall coherence. From a CLT perspective, collaborative learning may support learners in managing the cognitive demands associated with these interacting elements. Kirschner et al. [75]. asserted that effective collaborative learning involves a collective working memory that otherwise remains inactive. This collective working memory emerges through communication and coordination among group members, creating a shared cognitive space for learning. Within such a learning environment, learners can draw upon one another’s knowledge, clarify uncertainties, and jointly address writing-related challenges. As a result, collaborative engagement may distribute the processing demands associated with complex writing tasks and reduce extraneous cognitive load arising from confusion, fragmented understanding, or difficulties in interpreting task requirements.

Peer feedback can also be understood through the lens of CLT. Feedback activities require learners to revisit, evaluate, and discuss writing in relation to established criteria. Such engagement creates opportunities for reflection on both the feedback received and the feedback provided. Van Merrienboer and Ayres [52] argued that schemata become automated through repeated and successful application. In this sense, peer feedback, particularly when guided by explicit criteria and rubrics, may contribute to germane cognitive load because learners repeatedly apply evaluative standards while reviewing and revising written texts. The collaborative nature of peer-feedback activities may further support learners in recalling rubric criteria, clarifying uncertainties, and negotiating interpretations of writing quality. Through these processes, collaborative writing and rubric-guided peer feedback in the present study can be interpreted, from a CLT perspective, as instructional practices that support the management of intrinsic cognitive load, reduce unnecessary extraneous cognitive load, and facilitate schema construction and refinement in persuasive essay writing.

### The study

This phenomenological study aimed to explore how first-year undergraduates experienced the instructional design and materials (see ‘Methods’ section) devised for an English foundation course at the focal university while learning persuasive essay writing in an online environment. Guided by the following question, the study explored the lived experiences of the pupils concerning learning persuasive essay writing.

- How did first-year undergraduates experience and interpret the instructional design and materials used in the English foundation course in relation to their learning of persuasive essay writing during online teaching and learning?

## Methods

### The context and the researchers

Various studies have reported that difficulties in academic writing constitute a global challenge for students. In Bangladesh, these challenges are often compounded by limited instructional support and constraints in writing pedagogy. Consequently, supporting students’ development of academic writing remains an important responsibility of higher education institutions. Like other educational institutions, ‘Kiyan University’ kept it at the fore of its responsibility and established a separate and independent language institute named ‘Mumtahina Institute of Languages (pseudonym)’ that was equipped with expert and trained English language teachers to teach English foundation courses to first-year undergraduates. The first and second authors taught one such English foundation course during the Fall semester of 2020. They harnessed the instructional materials and resources prepared by the teachers at the language institute and cascaded them in the OTL platform ‘Arwa’. Persuasive essay writing was one of the course components for which they collaborated with other teachers to design instructional materials and subsequently provided instruction. This experience motivated them to undertake a study exploring how the instructional design and materials presented in ‘Arwa’ were experienced by students learning persuasive essay writing during the pandemic. At this juncture of the study, the first and second authors invited the third and fourth authors to integrate their expertise in research related to academic writing and ICT in education into this study. Given our dual role as instructors and researchers in this context, it is important to clarify how positionality was managed throughout the study.

### Reflexivity and researcher positionality

It is acknowledged that we were also instructors of the course in which the phenomenon occurred. This dual role may introduce potential interpretive bias as well as perceived power asymmetry between instructor and student participants. As qualitative methodologists note, researcher positionality inevitably shapes both data generation and interpretation and therefore requires deliberate reflexive engagement [76,77]. Given that we collected data through semi-structured interviews and open-ended questionnaires and were directly involved in the design and implementation of the persuasive essay curriculum in the OTL platform ‘Arwa’, we remained attentive to how our instructional positioning might influence participants’ responses and our subsequent interpretations.

Participation in the interviews and questionnaire was entirely voluntary. We informed participants that their academic evaluation was independent of their decision to participate, and we assured them that participation or non-participation would not influence their course outcomes [78]. During the interview process, we encouraged participants to articulate their experiences openly, including both affirming and critical reflections on the instructional design, collaborative writing activities, and rubric-guided peer feedback practices.

In the interpretive phase, we engaged in continuous reflexive awareness of our instructional involvement while analyzing the data. Following phenomenological principles that prioritize attentiveness to lived experience and the interpretation of meaning grounded in participants’ accounts [79], we revisited interview transcripts and questionnaire responses iteratively to ensure that emerging themes were grounded in participants’ descriptions rather than in our prior pedagogical expectations. This reflexive stance was sustained throughout the analytic process to support interpretive integrity.

### Online instructional context and course delivery

‘Arwa’ was Kiyan University’s OTL platform, built on a world-class platform developed by MIT and Harvard. It is like Coursera, Udemy, edX, etc. During the pandemic, when physical classes were impossible, ‘Arwa’ provided as near-live class experience as possible. The contents of all the courses offered in the focal university were uploaded on ‘Arwa’. Students were instructed to go through each week’s course content uploaded on ‘Arwa’ and complete the activities within the deadline set by the course instructors. The university did not allow students to rely solely on ‘Arwa’ for knowledge acquisition. In addition to their enrollment in ‘Arwa’, the students were also advised to attend the two weekly live sessions for a course, each for 1 hour and 20 minutes. In the live sessions, they had the opportunity to have their queries resolved by the concerned faculty members through rigorous interactions. The following table (Table 1) illustrates the nature of the support of the focal university for its students during the pandemic.

**Table 1:** The support system undertaken by the focal university during the pandemic.

The mode of transferring knowledge	The online platform used to transfer knowledge	The people involved in preparing the instructional materials	Additional support for the students
Video	‘Arwa’	The teachers and staff	Live sessions conducted by the concerned faculty members of the courses
Handout			

The English foundation course was delivered through a structured sequence of online learning activities in the OTL platform ‘Arwa’. Students first engaged individually with instructional materials uploaded to the platform and then participated in synchronous live sessions, collaborative writing tasks, and rubric-guided peer feedback activities. This sequence enabled them to move from initial exposure to persuasive essay components toward guided practice, collaborative drafting, peer review, and revision.

The English foundation course was delivered through a combination of synchronous and asynchronous online learning practices, which are widely documented in the literature [80]. In asynchronous online teaching, materials, resources, and announcements are disseminated, teacher-made videos are shared, space for written interactions in the discussion forum and assignment submission zone are provided, examinations are taken, and marks and feedback are shared using a learning management system (LMS), whereas, in synchronous online teaching, students meet their teachers and peers in real-time through the means of video-conferencing software [80]. Both asynchronous and synchronous online teaching and learning were conceptualized to teach the English foundation course at ‘Kiyan University’ [81,82]. ‘Arwa’ was used to share teacher-made instructional materials, instructions, and announcements, conduct examinations and inform scores, and provide written feedback to the students of the English foundation course. In addition, weekly live sessions were conducted where students met their teachers and peers, clarified their confusion, and asked for any support they needed. Hence, the synchronous and asynchronous forms of sharing knowledge online were instrumental to the successful outcome.

### Structure of the online instructional design

To enhance clarity of the instructional context, Table 2 presents a structured overview of the course design, including the sequence of weekly topics, the associated instructional materials, and the learning activities implemented in the OTL platform ‘Arwa’. Briefly, it shows the sequence of modules, the instructional materials used at each stage, and the progression from orientation to essay components, guided practice, collaborative writing, and peer feedback.

**Table 2:** Overview of instructional design and learning activities in the OTL platform.

Week-wise distribution of learning materials	Modes of instilling the learning materials
**Week 1:** Acquainting Students with Academic and Persuasive Essays	**Lecture Video:** Intended to introduce students to academic and persuasive essays and give the students a basic understanding of academic and persuasive essays. **Handout:** Offered the definition and structure of academic and persuasive essays. **Online Teaching:** Two days on a weekly basis, the teachers appeared in live sessions in which they tended to respond to the students’ queries related to the video and handout.
**Week 2:** Introducing the writing process of a thesis statement	**Lecture Video:** Containing the theoretical definition of ‘thesis statement’ and its practical implications. An example of a thesis statement in a deconstructed form has been presented. ‘Dos’ and ‘Don’ts’ to be considered while writing a thesis statement were highlighted. **Handout:** Students were provided with the handout that contained the definition of the thesis statement, the examples of deconstructed versions of the thesis statements coupled with the explanation, Dos, and Don’ts to be considered while writing a thesis statement. **Worksheet:** Students were presented with the worksheet. Before the students started writing a thesis statement on a given topic, they were supplied with adequate examples that guided them to develop a correct thesis statement. **Online Teaching:** During the weekly live sessions, the teachers lectured and supported them about the process of writing a thesis statement. The teachers also gave them feedback on their thesis statements on the worksheets.
**Week 3:** Introducing students to the ‘outline’ and ‘the processes of writing an *introduction* for a persuasive essay	**Lecture Video:** Informed about the *outline* and process of developing an *introduction* – A clear deconstruction of an introduction was shown in which the ‘grabber’, ‘connecting ideas’, and ‘thesis statement’ were highlighted, along with emphasizing the use of transitional words and *parallel structure* to write a thesis statement. Finally, it ended by reporting some ‘*Dos*’ and ‘*Don’ts*’ to be remembered while writing an introductory paragraph. **Handout:** With an illustrative form, it was a written note of what was discussed in the lecture video. **Online Teaching:** The teachers appeared in live sessions in which they checked the understanding of the students about the lesson and clarified their queries.
**Week 4:** Introducing grabber.	**Lecture Video:** Informing students about five types of *grabbers* with more explanation and examples. **Worksheet:** A worksheet was prepared in which students were asked to write effective grabbers on the given topics. **Group activity:** The students were instructed to form groups, each consisting of five members. They were specifically instructed to develop an outline and write an introduction on the topics assigned to them, provided that each group was assigned a particular topic to complete a persuasive essay. They were instructed to write in *Google Docs* so the teacher could monitor their progress. **Online Teaching:** Live sessions with teachers were dedicated to discussing, clarifying, or explaining anything related to the grabber, activity worksheets, and group work.
**Week 5:** Introducing the components of body paragraphs for writing a persuasive essay	**Lecture Video:** Presenting *topic sentences*, *supporting sentences* (*explanation* and *evidence*), and concluding sentences, coupled with the demonstration of deconstructed body paragraphs and some useful tips for the students to develop effective body paragraphs. **Handout:** The illustration of a deconstructed body paragraph. Besides, the types of evidence were conspicuously illustrated to inform students about the process of writing supporting sentences. **Reinforcement:** The lecture also emphasized the use of *transitional words* along with examples to write *explanations* and provide *evidence*. **Online Teaching:** Live sessions with teachers were dedicated to discussing, clarifying, or explaining anything related to developing body paragraphs. Also, they checked the extent to which students galvanized their understanding of the topic being taught.
**Week 5:** Introducing the components of a *conclusion*	**Lecture Video:** It informed the structure, including the restatement of the thesis statement, making suggestions or comments, and writing a clincher of a conclusion. It embedded the demonstration of restating the thesis statement, making suggestions, and developing a clincher. The deconstruction of a conclusion was verbally presented. **Handout:** The deconstruction of a conclusion was visually presented. In addition. students were shown how a grabber on a given topic led to the inclusion of a clincher. **Individual activity:** A worksheet was developed for students. It contained five activities dedicated to the practice of restating the thesis statement, and specific tasks were designed to involve students in writing clinchers. In the worksheet, a grabber on a topic was given. They were asked to write the clincher following the given grabber. **Group activity:** Finally, based on the elements taught, the students were asked to complete writing *body paragraphs* and *conclusions* on the same topics on which the groups drew *outlines* and wrote *introductions*. **Online teaching:** During the weekly live sessions, teachers asked questions to check students’ understanding, discussed relevant things, provided instructions concerning the activities, and clarified their confusion. Since the activities were to be done in *Google Docs*, they also checked whether all the students participated. If needed, they reminded those who were reluctant to accomplish the activities.
**Week 6:** Orientation to the checklist for peer checking and peer feedback.	**Handout:** It contains a checklist for assessing their peers’ persuasive essays. **Instructions:** Once the groups finalized their writings, they were asked to share their essays in Google Docs with other groups, as the concerned faculty member(s) assigned. For example, to generate peer feedback, Group A checked Group B’s essay, and Group B checked Group A’s essay. They were asked to follow the checklist (Table 3). After reading the essays and noting the feedback, the groups were asked to verbally deliver the feedback in front of the concerned faculty member. While sharing feedback, the groups were advised to highlight the ‘did well’ part first and then the ‘could improve’ part. They were encouraged to share the screen to visually present the areas where their peers could improve and advise how these could be improved. **Teachers’ role during the live sessions:** The teachers acted as moderators for activating groups to provide their peers with feedback.

Peer feedback was conducted as a structured group activity. After completing their drafts in Google Docs, each group exchanged essays with another group assigned by the instructor. Students used the checklist in Table 3 to evaluate the outline, introduction, body paragraphs, and conclusion of their peers’ essays. They first identified strengths (did well) and then areas for improvement (could improve), after which they delivered feedback verbally during the live session in the presence of the instructor. In this collaborative online process, instructors designed the materials, organized group exchange, moderated the live feedback sessions, and monitored participation, while students engaged with the materials, co-constructed drafts, evaluated peer work using the checklist, and revised their essays accordingly.

**Table 3:** Rubric-guided checklist for peer feedback on persuasive essays.

Components of a Persuasive Essay	Checklist	Sample peer feedback
Outline	• Is it a complete outline? • Does it have sub-ideas and evidence written in bullet points? • Does it have a thesis statement, topic sentences and restatement of the thesis statement written in complete sentences?	The grabber is interesting, but more background information can be added in the connecting sentences. The thesis statement has 3 reasons; however, a parallel structure could be maintained for further clarity.
Introduction	• Does the Introduction have 3 parts- grabber, connectingsentences, and the Thesis Statement? • Is the grabber interesting? Is it relevant to the topic? Does itengage the reader? • Do the connecting sentences (background information)prepare the reader for the thesis? Are they adequatelydeveloped? • Does the thesis statement clearly express the main claimand the 3 reasons? Are the 3 reasons presented in a parallel structure?	
Body Paragraphs (Body Paragraphs 1, 2, & 3)?	• Does each body paragraph have a 3-part structure- a topic sentence, supporting sentences, and a concluding sentence? • Do the topic sentences of three body paragraphs express Reasons 1, 2, & 3 from the ThesisStatement? • Do the supporting sentences provide an adequateexplanation of the reason and supporting ideas? • Is there any evidence used? Is the evidence strong enough forthe reader? • Does the concluding sentence summarize this paragraphwell?	
Conclusion	• Does the conclusion summarize the essay by rephrasing the thesis statement with different wording? • Are the suggestions and comments relevant to the topic? • Does the clincher relate to the grabber in the introduction? • Has the conclusion avoided adding any new ideas or information?	

### Research design

The study aimed to explore how first-year undergraduates experienced and interpreted the instructional design and materials used to support persuasive essay writing in an English foundation course. It encompassed the learning experiences of the students who enrolled in that course during the Fall semester of 2020 at Kiyan University. They were aided by the instructional design and materials posted in the OTL platform ‘Arwa’. We adopted a phenomenological study since it enabled us to explore the phenomenon (i.e., online teaching of persuasive essays through instructional design and materials posted in ‘Arwa’) experienced by a group of students. Creswell and Poth [78] advocate that a phenomenological study focuses on a phenomenon explored by a group of individuals who have all experienced the phenomenon. Van Manen [83] identifies that the experience of learning is a phenomenon to be researched following the phenomenological approach. This phenomenological orientation was considered appropriate because the study sought to understand how students experienced the instructional design, collaborative writing, and peer feedback processes in online persuasive essay learning, rather than to measure predefined instructional outcomes.

Specifically, we adopted an interpretive phenomenological approach [84] using semi-structured interviews and an open-ended questionnaire to explore participants’ understandings and experiences of persuasive essay writing following their engagement with the instructional materials, collaborative writing activities, and peer-feedback practices incorporated into the course. The instructional design implemented in the course is treated as a naturally occurring pedagogical context rather than an intervention to be evaluated, as the study focuses on participants’ lived experiences rather than causal effects. The staged data-collection process was aligned with this interpretive phenomenological orientation. Within interpretive phenomenology, experiences are understood as unfolding through individuals’ engagement with a phenomenon and acquiring meaning through reflection and interpretation [78,84,85]. Accordingly, the two interview phases were not designed to examine learning gains through a pre- and post-instructional comparison. Instead, they enabled participants to reflect on their experiences of persuasive essay writing at different points of engagement with the phenomenon. Exploring participants’ prior and subsequent understandings of persuasive essay writing provided insight into how they experienced, interpreted, and made sense of the instructional materials, collaborative writing activities, and rubric-guided peer feedback throughout the learning process [78]. We found this approach appropriate because “it is concerned with lived experiences and seeks reality in individuals’ narratives of their experiences of and feelings about specific phenomena, producing their in-depth descriptions” [86]. The analysis followed a phenomenological thematic analysis informed by Van Manen’s [85] hermeneutic phenomenological framework, focusing on interpreting participants’ lived experiences through iterative thematic engagement with the data.

### Participants

Eight hundred fifteen students enrolled in the English foundation course in the Fall semester of 2020. They belonged to different departments, including Architecture, Computer Science and Engineering, Economics and Social Sciences, Electrical and Electronic Engineering, Mathematics and Natural Sciences, and Pharmacy, doing their Bachelor of Architecture, Bachelor of Science in Computer Science, Bachelor of Social Science in Economics and Anthropology, Bachelor of Science in Electrical and Electronic Engineering, and Electronic and Communication Engineering, BA in English, Bachelor of Science in Applied Physics and Electronics, Mathematics, Physics, Biotechnology and Microbiology. During their admission test, their English (reading, writing, and speaking) proficiency were assessed, and based on their proficiency level (elaborately defined in Table 4), they had to enroll in the English Foundation Course in the first year. They were taught following instructional design and materials presented in ‘Arwa’.

**Table 4:** The details of the participants.

Participants (Pseudonyms)	Gender	Year and Semester at ‘Kiyan University’	Pre-university education: Type of institution	Exposure to online teaching and learning in earlier education	Proficiency level of English Writing of the Students enrolling in the English Foundation Course
S1	Male	Second Semester, First Year	Bangla Medium Institution	No	Although the students were not able to structure academic essays properly, they were capable of addressing the topic adequately with the development of at least one central idea. They were able to develop the main points well-supported with explanations and examples. They tend to write complete simple, complex and compound sentences that are mostly free from grammatical errors, such as subject-verb agreement and verb tenses. They attributed the quality to using a good range of vocabulary, although not varied.
S2	Male	”	”	No	
S3	Female	”	”	No	
S4	Male	”	English Medium Institution	No	
S5	Female	”	”	No	
S6	Male	”	Bangla Medium Institution	No	
S7	Male	”	”	No	
S8	Female	”	”	No	

The first author mentored two sections, and the second author mentored three sections (each section consisted of 32 students). Hence, among 815 students, 160 were connected to them. As interpretive phenomenology seeks participants who have directly experienced the phenomenon under investigation, we employed criterion sampling to ensure that all participants had engaged with the same instructional design, collaborative writing activities, and rubric-guided peer feedback practices implemented in the OTL platform ‘Arwa’ [78]. The inclusion criteria were therefore intentionally bounded. To illustrate, participants had to (a) complete the English foundation course in Fall 2020, (b) experience the specific instructional design and materials devised for persuasive essay writing, and (c) participate in collaborative writing and peer feedback activities structured within that course. This homogeneity was sought to preserve phenomenological coherence, as variation in instructional exposure could dilute the depth of experiential analysis [79].

Although 160 students were accessible to us, phenomenological inquiry prioritizes depth of experiential understanding rather than breadth of representation; therefore, we selected eight information-rich participants whose accounts could support intensive interpretive analysis [79]. Considering Creswell’s [87] suggestion that a phenomenological study may include three to ten participants, we purposefully selected eight participants from the accessible cohort. The final selection was guided by participants’ willingness to articulate detailed reflections on their experiences, thereby enhancing the richness of the data rather than seeking statistical representativeness. Data collection continued until thematic saturation was reached, that is, no new substantive themes emerged from the data. As such, the sample size was considered sufficient for interpretive phenomenological inquiry, which prioritizes depth of experiential understanding over numerical representation [78,79].

While the study involved a large initial cohort (N = 815), interpretive phenomenology does not aim for statistical representativeness but rather seeks to capture the essence of a shared experience through in-depth accounts. The selected participants adequately represent the phenomenon in that all of them engaged with the same instructional design, collaborative writing activities, and rubric-guided peer feedback practices within the OTL platform ‘Arwa’. This shared experiential context ensured that their accounts reflected the core features of the phenomenon under investigation. However, we acknowledge that transferability of the findings may be constrained by both the small sample size and the institutional context in which the study was conducted. The participants were drawn from a technologically supported private university environment and had relatively stable access to online learning resources, conditions that may not be available in less-resourced educational settings. In line with qualitative research principles, we provide rich, contextualized descriptions to enable readers to determine the applicability of the findings to similar educational settings [88].

The participants had no exposure to pair work and or group work to accomplish writing paragraphs, essays, or compositions during their pre-university education. Generally, the practical exposure of the students to collaborative work occurs at the tertiary level in Bangladesh, especially during teaching and learning of EAP courses [40]. None of them was engaged in providing peer feedback in their earlier stages of education. This relative lack of prior collaborative and peer feedback experience allowed the study to explore how structured collaborative writing and rubric-guided peer feedback were perceived when introduced within the online instructional environment. Table 4 contains the details of the participants.

In a Google Meet meeting, we shared the idea of the study with the founder and the then director of the ‘Mumtahina Institute of Languages’ (Pseudonym). Additionally, we informed the then Vice President for Research about the presentation of the instructional design and materials to serve the purpose of the study. We informed the director and the Vice President for Research about the proposed study and received their administrative support to proceed with the approval process. Nevertheless, the director instructed us to submit the Research notification form to the language institutes’ steering committee, which has been established by taking consent from the university’s central Research Metrics Committee (RMC). We filled out the form and submitted it to obtain ethical approval from the Mumtahina Institute of Languages’ Steering Committee for Research Review, the body responsible for reviewing research involving human participants within the institute. The study received ethical approval from the Mumtahina Institute of Languages’ Steering Committee for Research Review (Approval Reference No. BIL/BSC/001/2020) prior to data collection. Following this approval, a written invitation letter was issued to the students to participate in the study. The letter contained their rights to withdraw from the study, the details about the purpose of the study, the confidentiality to be maintained, and the benefits of the study [78].

### Data collection

We conducted multiple interviews with the same individuals and administered an “open-ended questionnaire” [78]. Before commencing the lessons on persuasive essay writing, we interviewed participants to explore their prior understandings of persuasive essay writing. Hence, the interview questions explored participants’ prior understandings of the structure and different components, e.g., introduction, body paragraphs, and conclusion, of a persuasive essay before enrolling in the course. In phase 2, after the completion of the lessons, we explored participants’ subsequent understandings of persuasive essay writing following their engagement with the instructional materials and learning activities. For this to be accomplished meaningfully, the interview questions in phase 2 were compartmentalized based on the way instructional materials were organized and presented in the OLT platform ‘Arwa’. For instance, lecture videos and handouts on academic and persuasive essays were presented to them in the first phase. Accordingly, we formulated the first interview question to explore participants’ understandings of persuasive essay writing after watching lecture videos, reading handouts, and receiving direct mentorship from teachers during weekly live sessions. The remaining interview questions were similarly developed to explore participants’ understandings of the structure and different components of a persuasive essay following their engagement with the instructional materials and activities. We later utilized these accounts, together with participants’ written reflections in the open-ended questionnaire, to understand how they experienced and interpreted the instructional design and materials used in the course. The average session of interviews with each participant in both phases was 25–30 minutes. We recorded the interview on a Samsung J7 Max mobile device.

In the final phase, we administered the open-ended questionnaire to learn the participants’ overall experiences of learning through online instruction, which was guided by instructional design and materials presented in ‘Arwa’, collaborative writing, and peer feedback on their persuasive essays. We used the open-ended questionnaire as a supplementary qualitative instrument to capture written reflections that participants might articulate more fully outside the interview setting and to enrich the phenomenological understanding of their experiences. In qualitative inquiry, open-ended questionnaires can function as a complementary source of narrative data when they are used to elicit participants’ own words rather than fixed-response patterns [78]. In addition, the questionnaire provided opportunities for participants to reflect on the potential relevance of online teaching and learning beyond the immediate pandemic context and in the event of future disruptions to face-to-face education. The use of two qualitative data sources, e.g., semi-structured interviews (Phases 1 and 2) and a supplementary open-ended questionnaire (final phase), enabled us to compare participants’ accounts across sources during analysis, thereby supporting source triangulation as a credibility-enhancing strategy in qualitative inquiry [78,88].

### Data analysis

We first transcribed the interview data and combined the responses to the open-ended questionnaire, which resulted in eight sets of raw data for the study. The analysis process was recursive, and both researchers were actively involved in going back and forth with the data to interpret meaning. We discussed emerging interpretations throughout the analytic process and resolved differences through repeated engagement with the data and interpretive discussion. We adopted Van Manen’s (85) three approaches for isolating themes. In the detailed reading approach, we examined each participant’s raw data closely to identify meaning units relevant to the phenomenon. In the selective or highlighting approach, we highlighted significant words, phrases, and statements, pasted and tabulated them in an MS Word document, and clustered them into concepts and sub-themes. In the holistic reading approach, we examined each account as a whole and used the sub-themes that recurred across participants to construct the final themes. Initial codes were generated inductively from participants’ expressions and meaningful statements identified during the detailed and selective reading stages. These codes were subsequently grouped into conceptual categories, which were refined into sub-themes and later synthesized into overarching themes through iterative comparison across participants’ accounts and continuous movement between parts and the whole of the data. This process of moving from raw data to thematic interpretation is illustrated in Table 5. Following the development of the preliminary thematic structure, we conducted member checking by inviting all eight participants to review selected excerpts from their accounts together with the corresponding thematic interpretations. Participants confirmed that the interpretations reflected their experiences of learning persuasive essay writing through the instructional design and materials implemented in the OTL platform ‘Arwa’, and no substantive revisions to the thematic interpretations were requested.

**Table 5:** Illustrative example of theme development from raw data.

Excerpts	Keywords/Meaningful Phrases	Sub-themes/Concepts	Final Theme
I wrote argumentative essays at the O level and learned the pattern of an academic essay, but I did not learn persuasive essay writing specifically.	Argumentative essays; O level; Pattern of an academic essay; lack of specific prior instruction in persuasive essay writing	Prior experience with essay types (argumentative); Knowledge of general academic essay structure; Lack of specific training in persuasive writing	Prior Knowledge of Persuasive Essay Writing
I wrote descriptive and narrative essays at my O Level. So, I have a prior idea about academic essays. I knew about the structure of an essay.	Descriptive and narrative essays; O level; Prior exposure to academic essays; Knowledge of essay structure	Prior experience with essay types (descriptive, narrative); Familiarity with general essay structure	
I did write essays before, but I did not follow any structure. I was not taught this earlier.	Did not follow any structure; Not taught this earlier	Lack of structured writing practice; Absence of prior instruction	
I was unaware of the structure of an academic essay before this course.	Unawareness of academic essay structure; No prior instruction	Limited prior knowledge of essay structure; Lack of formal instruction	

An external reviewer, who was an experienced qualitative researcher and applied linguist familiar with qualitative inquiry and English for Academic Purposes pedagogy in Bangladeshi private universities, reviewed the coding framework, thematic clustering, and interpretive rationale to examine the coherence between the data and the emerging themes. Feedback from this review informed further refinement of the thematic structure. We also engaged in peer debriefing through discussions within the research team, which enabled us to critically examine emerging interpretations, question assumptions, and refine the conceptual boundaries of the themes.

To enhance transparency of the analytic process, Table 5 provides an illustrative example of how raw data excerpts were transformed into keywords or meaningful phrases, which were then analytically reduced and grouped into sub-themes or conceptual categories. These sub-themes were subsequently synthesized into overarching themes through iterative comparison across participants’ accounts. The table demonstrates this inductive progression by showing how the first theme, i.e., prior knowledge of persuasive essay writing, was developed from participants’ narratives through inductive and interpretive analysis.

### Trustworthiness

To enhance the trustworthiness of this interpretive phenomenological study, we drew on the criteria of credibility, dependability, and confirmability proposed in qualitative inquiry [88]. Consistent with phenomenological principles that prioritize attentiveness to lived experience [79], we sought to ensure that interpretations remained grounded in participants’ narratives rather than in abstract generalizations.

Credibility was supported through sustained engagement with the data and member checking, which enabled participants to validate the resonance of the thematic interpretations with their lived experiences [78,88]. All eight participants were invited to review selected excerpts from their accounts together with the corresponding thematic interpretations. They were asked to determine whether the interpretations reflected their experiences of learning persuasive essay writing through the instructional design, collaborative writing activities, and peer-feedback processes. No participant requested substantive changes to the thematic interpretations. Peer debriefing further supported credibility through discussions within the research team that critically examined emerging interpretations, thematic boundaries, and the relationship between participants’ accounts and the developing themes [78]. Besides, dependability was strengthened through systematic documentation of the analytic process and through an external audit conducted by an experienced qualitative researcher and applied linguist familiar with qualitative inquiry and English for Academic Purposes pedagogy in Bangladeshi private universities. The reviewer examined selected interview transcripts, coding records, and thematic development documents to assess the coherence between the data, coding framework, and thematic interpretations [88]. Furthermore, confirmability was ensured by maintaining a transparent audit trail linking raw data, analytic decisions, and final thematic interpretations, alongside reflexive engagement with the data throughout the analytic process [79,88]. The audit trail comprised interview transcripts, open-ended questionnaire responses, coding records, and thematic development documents that documented the progression from participants’ excerpts to meaningful phrases and keywords, sub-themes, and final themes. Additionally, the use of two qualitative data sources, e.g., semi-structured interviews and open-ended questionnaires, enabled source triangulation, strengthening the credibility of the findings [88].

Because the study adopted an interpretive phenomenological orientation, we did not pursue statistical inter-coder reliability; instead, we ensured analytic rigor through collaborative interpretation, reflexive engagement, and external review. Interpretive phenomenology prioritizes coherence, depth of meaning, and transparency of interpretation over coder agreement metrics [78,79].

### Ethical considerations

We conducted this study in accordance with established ethical principles governing human participant research. Prior to commencing the study, we obtained approval from the Mumtahina Institute of Languages’ Steering Committee for Research Review (Approval Reference No. BIL/BSC/001/2020), which oversees and reviews research involving human participants. The committee reviewed the study to ensure that appropriate measures were in place to protect participants’ rights, confidentiality, and well-being. We informed participants about the purpose of the study, the procedures involved, and their rights as research participants, including their right to withdraw from the study at any stage without penalty. We clearly stated that participation in the study would not influence their course grades or academic standing. We obtained written informed consent from all participants prior to conducting the interviews and collecting questionnaire responses. Data were collected during the course; however, we took specific measures to minimize potential power imbalance between instructor-researchers and student participants. As we served as instructors of the course, we remained critically aware of the potential power imbalance between instructor-researchers and student participants and took deliberate steps to mitigate its influence on participation and responses. To address this, we clearly informed participants that their decision to participate or decline would have no impact on their course grades or academic standing, and we emphasized that participation was entirely voluntary. We also assured participants that their responses would not be used for evaluative purposes and would be accessed only after course-related assessment processes were completed. Besides, we maintained participants’ confidentiality throughout the study. Consequently, we assigned pseudonyms to all participants during transcription and used these identifiers in analysis and reporting. Besides, we stored interview recordings, transcripts, and questionnaire responses in password-protected digital files accessible only to the research team. We used the data solely for research purposes and did not disclose any identifiable personal information in publications or presentations. Such measures are consistent with qualitative research ethics, which emphasize voluntary participation, informed consent, and the protection of participants in contexts where researcher–participant relationships may involve asymmetry [78].

## Findings

The phenomenon that the current study explored was the lived experiences of the participants who learned persuasive essay writing through the online teaching and learning platform ‘Arwa’ during COVID-19. To understand these experiences, participants’ prior understandings of persuasive essay writing were explored before the commencement of the lessons. After engaging with the instructional materials and learning activities, participants reflected on their understandings of different components of persuasive essay writing, while the open-ended questionnaire provided opportunities for them to share their experiences of instructional materials, collaborative writing, and peer-feedback practices. The study also explored their overall experiences of learning persuasive essay writing online and their perspectives on the sustainability of such teaching initiatives beyond the immediate pandemic context. The findings are presented under four themes that emerged through phenomenological thematic analysis of the semi-structured interviews and open-ended questionnaire responses. They are reported as participants’ lived experiences, understandings, and perceptions rather than as objective measures of instructional effectiveness.

### Prior knowledge of persuasive essay writing

Participants’ prior understandings of persuasive essay writing were explored through their descriptions of the structure, introduction, body paragraphs, and conclusion of a persuasive essay. It became apparent that participants’ prior knowledge was highly varied, ranging from some structured understanding of general essay types to a complete lack of formal instruction.

#### Prior experience with general academic essays but lack of specific persuasive writing knowledge.

Some participants noted that they had prior knowledge about the structure of a general academic essay, which they had developed through exposure to other essay types in their O Level studies. However, this knowledge did not specifically extend to persuasive writing.

I wrote argumentative essays at the O level and learned the pattern of an academic essay, but I did not learn persuasive essay writing specifically. Hence, I did not have any knowledge of its exact structure, but in argumentative essays, I followed a structure: Introduction, Body paragraphs, and Conclusion. I am not sure if the same is applicable to persuasive essays. (S4)

I wrote descriptive and narrative essays at my O Level. So, I have a prior idea about academic essays. I knew about the structure of an essay. (S5)

These responses suggest that some participants had prior exposure to general essay forms, although this did not extend to persuasive essay writing specifically.

### Limited or no prior knowledge of essay structure

Some of the participants reported a complete absence of knowledge about academic essay structure. This was particularly evident among students from institutions following the National Curriculum and Textbook Board (NCTB) syllabus. Several participants from these educational backgrounds described limited exposure to structured essay writing before enrolling in the course.

I did write essays before, but not persuasive essays. I did not follow any structure. I was not taught this earlier. I did not have any idea about the structure of a persuasive essay. (S7)

I was unaware of the structure of a persuasive essay, let alone the structure of a persuasive essay. (S8)

These responses indicate limited prior familiarity with structured essay writing among several participants.

#### Mixed responses on prior knowledge of essay components.

Participants expressed mixed and often fragmented knowledge about the specific components of an essay, particularly for the introduction, body paragraphs, and conclusion. Regarding the introduction, some participants had prior, but superficial, knowledge, while others had none.

I wrote essays as part of creative writing processes. I wrote an eye-catching introduction. My previous teacher taught me to make it eye-catching. (S1)

I was taught to write an interesting introduction that can grab the reader’s attention, but it was not for a persuasive essay. In general, I learned to write an introduction like this. (S4)

I have no prior idea about the formation of an introduction. I just thought for a while and wrote. I started the introduction with random ideas that came to my mind. (S2)

These responses show that participants entered the course with varied prior understandings of essay introductions. While some had limited familiarity with attention-grabbing openings, others described writing introductions without clear structural guidance. Similarly, responses concerning prior knowledge of body paragraphs were mixed. A few participants demonstrated some understanding, while others had none.

I had a prior idea about the body paragraph. It starts with the topic sentence, includes relevant supporting details, and ends with a concluding sentence. (S2)

S3 voiced a similar response, confirming “the existence of prior ideas about the components, i.e., topic sentence, supporting details, and the concluding sentence of body paragraphs.” By contrast, other participants reported no prior knowledge about forming a body paragraph.

I used to write descriptions about the topic in the body paragraph. I used to give suggestions in the body paragraphs. (S4)

I used to form the body paragraphs in my way. I did not know what to write or how to write exactly in response to the topic. (S5)

The responses concerning body paragraph structure reflected considerable variation in participants’ prior understandings. Some participants described familiarity with components such as topic sentences, supporting details, and concluding sentences, whereas others reported writing body paragraphs without a clear understanding of how these elements should be structured or connected.

Advancing further, Participants’ prior knowledge of the conclusion was also explored, producing mixed responses.

I had a prior idea that a concluding paragraph is a short paraphrased paragraph of the essay’s ideas. (S1)

When I wrote the essay, I could not write the conclusion. I did not know how to write the conclusion. (S2)

I used to write the conclusion, but I did not know how to write it. I summarized the information from the body paragraphs in the conclusion. (S4)

It is apparent from the responses that the participants entered the course with uneven and often limited prior knowledge of persuasive essay structure and its components.

### Participants’ understanding of persuasive essay writing after engaging with ‘Arwa’ and Live sessions

Participants reported changes in their understanding of persuasive essay structure after engaging with the course. Their accounts suggest that they developed a clearer understanding of the components of essay introductions, body paragraphs, and conclusions.

#### Enhanced understanding of essay introduction.

Participants noted that they now understood the specific components required for a persuasive essay introduction. They reported that this newfound knowledge was a key takeaway from the instructional materials and sessions.

Now I am aware of a grabber, connecting ideas, and a thesis statement in an introduction. (S1)

Including a grabber, connecting ideas, and a thesis statement is my new learning. Now, I can develop an introduction properly to write, and it is a meaningful learning experience for me. (S4)

These excerpts show how participants described their understanding of the components of a persuasive essay introduction after engaging with the instructional materials.

#### Reported understanding of body paragraph structure.

Participants also described their understanding of body paragraph structure, particularly the role of topic sentences, supporting details, and concluding sentences. They were able to clearly articulate the purpose of each sentence, from the topic sentence to the concluding sentence.

The lecture videos and handout contained meaningful explanations. Eventually, I gathered the knowledge to start with the topic sentence. Then, I shall explain it using supporting details. Transitional words should be used. The body paragraph ends with a concluding sentence. Throughout the process, my writing should be coherent and fluent. (S2)

I learn to start with a topic sentence; then, I have to give reasons, which act as supporting details, and add the concluding sentence to end the paragraph. I also learn to avoid adding new ideas in the concluding sentence. (S6)

These voices of the participants suggest that they became more aware of the structure and function of body paragraphs after engaging with the instructional materials.

#### Understanding of how to construct a conclusion.

Participants reported that they developed a clearer understanding of how to write a persuasive essay conclusion, noting that they had learned new components and how to properly connect the conclusion to the rest of the essay.

It is divided into three minor parts that are a restatement of the thesis statement, which is the actual thesis statement with different wording. Then come some suggestions and the clincher. (S2)

The inclusion of a clincher is new to me. In conclusion, I used to write the concluding sentence and add new information, but did not know that the conclusion must be connected with the introduction. Meaning to say, I have to restate the thesis statement and add some suggestions and a clincher. (S4)

These responses indicate that participants perceived themselves as developing a more structured understanding of how to write the conclusion of a persuasive essay. S4’s account shows how the participant described a change in understanding, particularly regarding the need to connect the conclusion with the introduction and avoid adding new information.

The participants’ responses suggest a movement from limited or fragmented understandings toward a more structured understanding of persuasive essay writing. Their ability to describe specific components of essay introductions, body paragraphs, and conclusions indicates how they experienced and understood persuasive essay writing after engaging with the instructional materials and learning activities. Participants described the instructional materials and learning activities as helping them understand the structure and components of persuasive essay writing more clearly.

### Usefulness of collaborative writing, peer feedback, and learning outcome

The third theme that emerged from the data concerned participants’ perceptions of the usefulness of collaborative writing and peer feedback in supporting persuasive essay writing. Participants experienced collaborative writing and peer feedback as useful in identifying and correcting errors, reconsidering essay structure, and confirming their understanding of persuasive essay writing. The following sub-themes and supporting data illustrate these benefits.

#### Collaborative writing and peer feedback as tools for error correction.

Participants described collaborative writing and peer feedback as processes that facilitated discussion and helped identify and correct structural errors in their writing.

Engaging in collaborative writing was helpful. We had a discussion before writing the outline. I suggested some irrelevant points... but the other group members corrected me. Feedback from peers improved the quality of the outline of our persuasive essay, as our outline had some flaws. The way we wrote the reasons in the outline did not match the body paragraph’s sequence. Peer feedback helped us identify those mistakes, and we got a chance to correct them before the final submission. (S1)

Collaborative writing and peer feedback helped…Through peer feedback, we learned that topic sentences were not relevantly aligned with the thesis statement. Again, we collaboratively dealt with the comment and revised it to include a relevant topic sentence in alignment with the thesis statement. (S5)

Other participants similarly noted collaborative writing and peer feedback as helping them identify structural inconsistencies, correct errors, and reconsider the organization of their essays.

#### Peer feedback as a mechanism for structural improvement.

Beyond simple error correction, participants perceived peer feedback as helping them reconsider and refine the overall structure of their essays, from individual paragraphs to the entire essay outline. Participants described receiving specific suggestions from their peers concerning the development of body paragraphs. They also reported discussing peer comments and revising their writing in response to suggestions concerning the organization and relevance of their arguments.

We made a few mistakes in our body paragraphs. Our peers from another group gave us specific advice on where we needed to work on the body paragraphs. We discussed the comments, and we worked collaboratively to correct the mistakes. (S2)

Working in groups helped address the knowledge gap. Peer feedback helped us write relevant supporting details that are related to the topic sentence. Due to peer feedback, we managed to write the restatement of the thesis statement properly and add relevant suggestions. (S3)

Participants also reported using peer feedback when revising conclusion-related components, including the restatement of the thesis statement, relevant suggestions, and the clincher.

Peer feedback helped us write a relevant clincher in their conclusion. (S1)

We managed to write the restatement of the thesis statement properly and add relevant suggestions. (S3)

Other participants similarly reported that peer feedback helped them refine different components of their essays, including body paragraphs and conclusions, by improving the organization and relevance of their ideas. By and large, they experienced peer feedback as helping them revisit different components of their essays, including body paragraphs and conclusions.

#### Peer feedback as a confirmation of understanding.

Participants also noted peer feedback as a way of validation, helping them confirm their own understanding of the assignment’s requirements. For these participants, peer feedback was not limited to correction; it also helped them check whether their writing followed the expected structure of a persuasive essay.

It’s like two-step authentication. First, an individual’s understanding was tested through group discussion in collaborative writing. Second, peer feedback further authenticated the correctness of the outline and helped us understand if my group was on the right track or not in the process of writing a persuasive essay. (S2)

While my group completed the first draft of the essay, another group confirmed its correctness. They focused on what we ‘did well’ in the essay. This time, they did not elicit anything under the ‘could improve’, which was an indication of our understanding of persuasive essay writing. (S7)

Other participants similarly perceived peer feedback as useful because it helped them verify their understanding and gain confidence in their work. Their accounts suggest that peer feedback was experienced not only as a process of correction but also as a way of checking whether their essays met the expected structure of persuasive writing. Participants perceived rubric-guided peer feedback as useful because it provided structured criteria for reviewing and discussing their writing.

### The overall impression of ‘Arwa’, collaborative writing, and peer feedback to learn persuasive essay writing

The final theme concerned participants’ overall impressions of ‘Arwa’, instructional materials, collaborative writing, and peer feedback as part of their online persuasive essay learning experience. Participants described these activities as useful for identifying and correcting errors, reconsidering essay structure, and confirming their understanding of persuasive essay requirements.

#### Perceived usefulness of instructional materials.

Participants found the core instructional materials, which include lecture videos and handouts, to be a crucial resource for their learning. They noted that these materials were not only helpful but also easily accessible, which facilitated independent learning.

The lecture videos and handouts were useful and easily accessible. I used to watch and read these. Moreover, group work was much needed during COVID-19. It helped a lot. Besides, peer feedback was effective and helpful as it helped us improve our essay writing. (S1)

This excerpt from S1 reflects the participant’s perception of the usefulness of the instructional materials in supporting their understanding of persuasive essay writing. Other respondents also expressed a similar impression. Participants described the lecture videos and handouts as accessible resources that supported independent engagement with the course materials before and during collaborative activities. These responses suggest that participants viewed the different components of the course as interconnected and supportive of their learning.

#### Collaborative activities as a source of motivation and support.

The respondents reported that in the context of the COVID-19 pandemic, the collaborative and peer feedback elements of the course served a crucial motivational function. They found that these activities provided a sense of community and encouragement when they felt isolated.

Lecture videos and handouts were helpful because I could learn independently. During the COVID-19 days, I lost motivation. My group members motivated me to learn the content and get involved in the writing process. Eventually, I watched the videos and read the handouts  ... peer feedback allowed us to see concrete examples of what worked and what needed to be improved in our writing. (S2)

S2’s account shows that peer interaction was experienced not only as academic support but also as encouragement during the COVID-19 period. S2 described group members as a source of social and emotional support that encouraged engagement with the instructional materials. These responses indicate that participants experienced collaborative activities not only as pedagogical support but also as a source of encouragement during a challenging period. Other participants also echoed the same view, informing the motivational and supportive space through collaborative activities.

## Discussion

An important contextual feature of the findings concerns participants’ prior educational experiences. Several participants reported limited exposure to structured persuasive essay writing before entering the course, particularly those who had studied within educational settings where essay writing was often approached as a broader writing activity rather than through explicit instruction concerning rhetorical structure and component parts. This background may help explain the uneven prior understandings reported by participants and provides context for interpreting their subsequent experiences of the instructional materials and learning activities.

The findings suggest that the progression from fragmented to more structured understanding of persuasive essay writing reflects a shift in how learners cognitively organize writing knowledge, rather than merely acquiring isolated skills. This transition may be interpreted as reflecting participants’ development of schemata related to essay structure, as described in their accounts of learning persuasive essay writing. From a Cognitive Load Theory perspective, this structured progression may be interpreted as indicating how participants gradually engaged with increasingly interconnected elements of persuasive essay writing [51,52]. Participants’ descriptions of their learning experiences suggest that the instructional design supported their understanding of relationships among different components of persuasive essay writing. This interpretation is consistent with prior research demonstrating that structured instructional support and guided writing practice enable learners to organize ideas more effectively and produce higher-quality academic writing [73,89]. The significance of this finding lies in the insight it provides into how participants experienced and interpreted the development of persuasive essay writing knowledge within an EMI online learning environment. Participants’ accounts suggest that the presentation of the learning contents on ‘Arwa’ was experienced as helpful in understanding persuasive essay writing. For instance, the teachers avoided presenting unrelated and scattered content when acquainting students with academic and persuasive essays through lecture videos, handouts, and worksheets. To illustrate, the learning content started with the definition of ‘Academic Essay’, followed by an elaboration on its structure. Participants’ accounts suggest that familiarity with the general structure of academic essays helped them engage with the structure of persuasive essays introduced later in the course. Van Merrienboer and Ayres [52] advocated that for enabling students to embrace and comprehend new content(s), the prime focus of the material designers or teachers must be ordering or organizing the instructional materials in a way that would enrich the schemata which are responsible for regulating interacting elements.

The introduction of thesis statement instruction through multiple formats, including video, handout, and worksheet, may be understood as providing learners with repeated opportunities to engage with thesis statement instruction across different formats. Rather than encountering the concept only once, learners encountered thesis statement instruction across different modalities, providing repeated opportunities to work with this component of persuasive essay writing. From a Cognitive Load Theory perspective, such repeated and structured exposure enables more efficient processing in working memory, as familiarity with the content reduces cognitive effort and facilitates quicker retrieval of relevant information [52]. In addition, the inclusion of practice through worksheets allowed learners to actively apply their understanding, further consolidating their knowledge. Participants’ accounts suggest that repeated engagement with thesis statement instruction supported their understanding of this component of persuasive essay writing [53].

The progression from introducing essay outlines to developing introductions and specific components, such as grabbers, can be interpreted as a scaffolded instructional sequence that gradually increases the complexity of writing tasks. Cognitive Load Theory proposes that instructional materials should be organized in ways that help learners process interacting elements without overwhelming working memory [52]. In the present study, participants encountered persuasive essay writing through a sequence that moved from understanding the overall structure of the essay to engaging with individual components before integrating them into larger writing tasks. Such sequencing may have supported learners in establishing relationships among different essay components while gradually expanding their understanding of persuasive essay writing. Participants’ accounts suggest that this progression helped them make sense of how introductions, body paragraphs, and conclusions functioned as interconnected elements of a persuasive essay. The significance of this finding lies in showing how online writing instruction in an EMI context may support learners’ engagement with complex writing tasks when instructional materials are organized as a coherent sequence rather than as isolated learning resources [52,53].

Moreover, involving the groups in accomplishing the activities can be interpreted in light of collaborative learning in relation to Cognitive Load Theory. The concept of collaborative learning relevant to this study concerned the involvement of five students in each group in achieving a learning goal by being supported by computers and internet access [50]. In reporting the benefit of continuing teaching and learning online, Al Fraidan and Al-Harazi [90] showed that collaborative learning caters to the clarification of learners’ queries and may create conditions that support learners’ engagement with writing-related activities. In the present study, specific tasks were assigned to the groups with the shared aim of writing an introduction, where instructions were clearly articulated to guide learners’ engagement with the task [50]. The use of shared digital platforms (e.g., Google Docs) enabled group members to collaboratively construct and revise their writing in a coordinated manner. In this regard, the increasing use of digital technologies in higher education has been shown to support collaborative writing practices that support learners’ engagement with collaborative writing processes [55–57,91]. Collaborative writing tasks that require higher levels of cognitive engagement can be effectively accomplished through such shared environments [58]. From a Cognitive Load Theory perspective, collaborative engagement in these tasks has implications for both intrinsic and extraneous cognitive load, as group members are able to distribute cognitive demands and support each other in processing complex writing components [50]. In this study, the assignment of structured group tasks may be interpreted as enabling learners to collectively engage with the interacting elements involved in writing an introduction, such as inserting an effective grabber, organizing connecting ideas, and developing a coherent thesis statement. This suggests that collaborative writing functioned not only as a pedagogical strategy but also as a cognitive support mechanism, allowing learners to engage with the demands of writing through shared interaction and discussion.

The subsequent stage of instruction, which focused on developing body paragraphs and conclusions, can be interpreted as extending learners’ engagement with more complex components of persuasive essay writing. In addition to lecture videos and handouts, the inclusion of live sessions enabled learners to clarify their understanding, negotiate meaning, and seek immediate support, thereby reinforcing their learning in an interactive environment. Similar to earlier stages, learners continued working collaboratively to construct body paragraphs and conclusions, which required them to generate, organize, and refine ideas collectively. Collaborative writing ensures joint participation in writing tasks, encouraging learners to deliberately generate ideas and identify effective ways of presenting them [59]. In this process, learners not only contributed individual inputs but also engaged in reviewing, discussing, and refining each other’s ideas, which reflects a more interactive and dialogic approach to writing. The activation of collective working memory occurs when group members communicate and coordinate their knowledge of the writing process, allowing them to manage complex writing tasks more effectively through shared cognitive effort. Moreover, the earlier instructional attempts to familiarize students with processes such as mind mapping, drafting, editing, and revising appear to have supported this collaborative engagement [89]. As learners repeatedly engaged with these processes in a group setting, they were able to draw on shared knowledge and prior learning experiences, which supported collaborative engagement with writing tasks. In this sense, collaborative writing at this stage can be understood as both a pedagogical strategy and a cognitive mechanism that supported learners in managing the demands of extended writing tasks.

The final stage of the instructional process, which involved rubric-driven peer checking and feedback, can be interpreted as a structured mechanism through which learners evaluated, validated, and refined their writing. López-Pellisa et al. [55] showed that students prefer engaging in reciprocal peer review, as the process sustains their attention to writing tasks. Similarly, Yu and Lee [92] found collaborative feedback effective, while Alshuraidah and Storch [59] revealed that learners share more insights when collaboratively providing peer feedback. In the present study, peer feedback extended beyond surface-level correction, as learners actively identified issues in outlines, introductions, body paragraphs, and conclusions, and communicated these observations within their groups. Importantly, the feedback process enabled learners not only to detect errors but also to evaluate the alignment and coherence of their writing. For instance, learners identified mismatches between thesis statements and topic sentences, clarified structural inconsistencies, and confirmed whether their writing met the expectations of persuasive essay conventions. In this sense, peer feedback functioned as both a diagnostic and confirmatory process, allowing learners to validate their understanding while refining their written work. This aligns with Kerman et al.’s [72] findings, which highlight the role of peer feedback in improving argumentative writing through problem identification and revision, as well as with Gao et al. [63], who noted that peer feedback is conducive to the development of writing skills. Moreover, the immediacy and reciprocity of feedback within collaborative groups enabled learners to engage in iterative cycles of reflection and revision. As groups exchanged feedback, reflected on comments, and revised their drafts, participants described becoming more attentive to the organization and coherence of their persuasive essays. In this sense, peer feedback was experienced not only as a means of identifying problems but also as an opportunity to examine persuasive essay conventions and reconsider writing decisions through interaction with peers.

The most significant aspect of the peer feedback process in this study was the use of specific guidelines, which contributed to the meaningful production of peer feedback. The learners were supplied with a checklist containing targeted questions (see Table 3) to assess various sections (i.e., outline, introduction, body paragraphs, and conclusion) of their peers’ persuasive essays. This structured approach enabled learners to focus their attention on specific features of writing, allowing them to provide more precise and relevant feedback. In this regard, peer feedback was not left to individual interpretation but was guided through clearly articulated criteria, which supported learners in identifying structural alignment, coherence, and the overall effectiveness of their peers’ writing. This interpretation is consistent with previous research emphasizing the value of structured peer feedback and explicit evaluative criteria in supporting learners’ engagement with writing tasks [61,62,73]. Similarly, the checklist used in this study appears to have functioned as a guiding tool that enabled learners to systematically evaluate and revise their writing. Although peer feedforward was not explicitly emphasized, learners revisited their essays, collaboratively corrected identified issues, and refined their work, resulting in revisions that participants perceived as strengthening their persuasive essays [61]. Importantly, this finding contrasts with studies that have raised concerns about the effectiveness of peer feedback and highlighted challenges such as limited student capability or lack of meaningful engagement [55,64,67]. In the present study, participants described rubric-guided feedback as providing clear direction for evaluating and responding to peers’ writing. This suggests that the effectiveness of peer feedback may depend not only on the activity itself but on the degree of structure and guidance provided to learners during the feedback process.

Furthermore, the incorporation of live sessions created opportunities for both providers and recipients of peer feedback to share their thoughts and reflections simultaneously, thereby shaping a dialogic learning environment [74]. Such an environment enabled learners to clarify misunderstandings, negotiate meaning, and refine their ideas through immediate interaction, which participants described as making peer feedback more meaningful and useful. Er et al. [74] emphasized the importance of incorporating rubrics in peer assessment to help learners identify strengths and weaknesses in writing, a feature that was evident in the present study. From a Cognitive Load Theory perspective, engagement in peer feedback activities also contributed to the repetition and reinforcement of knowledge stored in schemata. As learners reviewed their peers’ work, provided feedback, and reflected on the comments received, they were required to recall and apply previously learned concepts related to persuasive essay writing. This repeated engagement with core writing components may be interpreted as reinforcing participants’ understanding of persuasive essay components through repeated engagement, enabling more efficient processing and integration of knowledge [52,53]. Participants also described the combination of rubric-guided feedback and opportunities for discussion as helping them interpret feedback and reconsider their writing decisions. The opportunity to access peers’ work, reflect on it, and discuss it in live sessions further supported a deeper level of engagement with writing tasks. This is similar to Wang [70] in that the use of rubrics in peer feedback helps learners better understand evaluation criteria and improve their writing accordingly. These patterns point to the potential value of designing peer-feedback environments that combine structure, interaction, and opportunities for repeated engagement. In this study, participants described rubric-guided feedback, collaborative dialogue, and iterative revision as contributing to their understanding of persuasive essay writing and their engagement with the writing process, suggesting the value of integrating structure and dialogue within online EAP writing instruction.

## Conclusion, limitation, and recommendation

The current study explored how first-year undergraduates experienced and interpreted the instructional design and materials used to teach persuasive essay writing in an English foundation course during COVID-19. Drawing on semi-structured interviews and an open-ended questionnaire, the study examined participants’ reported understandings and experiences of online teaching and learning, collaborative writing, and rubric-guided peer feedback in relation to persuasive essay writing. The findings suggest that participants perceived structured instructional materials, collaborative writing, and rubric-guided peer feedback as supporting their engagement with writing processes and the refinement of their understanding of persuasive essay writing. Their experiences were shaped by the institutional context in which the study was conducted, including access to technological resources, stable internet connectivity, and support through the ‘Arwa’ platform. As these conditions may not be present in all educational settings, caution is warranted when considering the applicability of the findings beyond the study context.

The study has several limitations. It was conducted with a small number of participants within a single institutional context, and therefore, the findings are exploratory and should not be generalized to a wider population. The participants represented a specific demographic group with relatively stable access to technological resources, which may not reflect the experiences of learners in different socioeconomic contexts. In addition, the findings are based on self-reported data collected through interviews and open-ended questionnaires, which may be subject to bias. Furthermore, the study did not incorporate a comparison between online and face-to-face instructional approaches, nor did it examine variables such as gender, prior peer learning experiences, attitudes toward collaborative writing, or previous exposure to online teaching and learning. In addition, the study did not include performance-based measures such as writing samples or rubric scores; therefore, the findings are limited to participants’ reported experiences rather than objective measures of learning outcomes.

Future research may examine similar instructional initiatives used in other components of English writing and may employ quantitative or mixed-method approaches with larger samples to investigate their effectiveness across different contexts. Comparative and longitudinal studies may also provide deeper insights into how instructional design, collaborative writing, and peer feedback influence learners’ writing development over time. In addition, future studies may examine how contextual factors, such as access to technology and institutional support, shape learning outcomes in online environments. Based on the findings of this study, institutions aiming to enhance online teaching and learning of writing skills may consider developing structured online platforms, focusing on intentional instructional design, providing teacher training and support, and encouraging collaborative and feedback-oriented learning environments.

## Supporting information

S1 File De-identified participants’ responses. This file contains de-identified qualitative responses from the participants, including materials related to the semi-structured interviews and open-ended questionnaire responses used in the study.
(PDF)
