# Supplementary material for: Teaching persuasive essay writing online to first-year undergraduates: A phenomenological study of instructional design and learning experiences
Source: PLoS One. 2026 Jul 17;21(7):e0353171. doi: 10.1371/journal.pone.0353171 (PMC13379122; doi:10.1371/journal.pone.0353171)
Supplement: S1 File — This file contains de-identified qualitative responses from the participants, including materials related to the semi-structured interviews and open-ended questionnaire responses used in the study. (PDF) [file pone.0353171.s001.pdf]

Participant 1 (S1)

Interview: Phase 1

1. What was your prior knowledge about the structure of a persuasive essay?

I did not have any knowledge about the structure of a persuasive essay, but I knew that an essay [in general] should consist of different parts, like an introduction, body paragraphs, and a conclusion. I did not have specific knowledge of the structure of a persuasive essay.

2. What was your prior knowledge about the formation of an introduction of a persuasive essay?

I wrote essays as part of creative writing processes. I wrote an eye-catching introduction. My previous teacher taught me to make it eye-catching.

3. What was your prior knowledge about the formation of body paragraphs of a persuasive essay?

In the body paragraphs, I briefly discussed the topic of the essay.

4. What was your prior knowledge about the formation of a conclusion of a persuasive essay?

I had a prior idea that a concluding paragraph is a short paraphrased paragraph of the essay's ideas.

Interview: Phase 2

1. What is your current knowledge about the structure of a persuasive essay?

Now, I am clear that a persuasive essay consists of an introduction, body paragraphs and conclusion.

2. What is your current knowledge about the formation of an introduction of a persuasive essay?

Now I am aware of a grabber, connecting ideas, and a thesis statement in an introduction.

3. What is your current knowledge about the formation of body paragraphs of a persuasive essay?

I know it starts with a topic sentence, which I would later explain with supporting details. In presenting supporting details, I have to use transitional words.

4. What is your current knowledge about the formation of a conclusion of a persuasive essay?

Now, I start with the restatement of the thesis statement. My teacher encourages me to paraphrase it. Then, I write some suggestions and end with a clincher.

Participant 2 (S2)

Interview: Phase 1

1. What was your prior knowledge about the structure of a persuasive essay?

I knew the structure of an academic essay, but I didn't exactly know about a persuasive essay's structure. What I knew, in an essay, some introductory sentences have to be written to introduce the topic, body paragraphs are there to discuss more about the essay, and some sentences are to be written at the end.

2. What was your prior knowledge about the formation of an introduction of a persuasive essay?

I have no prior idea about the formation of an introduction. I just thought for a while and wrote. I started the introduction with random ideas that came to my mind.

3. What was your prior knowledge about the formation of body paragraphs of a persuasive essay?

I had a prior idea about the body paragraph. It starts with the topic sentence, includes relevant supporting details, and ends with a concluding sentence.

4. What was your prior knowledge about the formation of a conclusion of a persuasive essay?

When I wrote the essay, I could not write the conclusion. I did not know how to write the conclusion.

Interview: Phase 2

1. What is your current knowledge about the structure of a persuasive essay?

I have a clear understanding of the structure: Introduction, Body paragraphs, and Conclusion.

2. What is your current knowledge about the formation of an introduction of a persuasive essay?

I developed a clear understanding of the introduction. I have to start with a grabber to attract the readers and add connecting sentences that would lead to the thesis statement, but I have to maintain coherence.

3. What is your current knowledge about the formation of body paragraphs of a persuasive essay?

The lecture videos and handout contained meaningful explanations. Eventually, I gather the knowledge to start with the topic sentence. Then, I shall explain it using supporting details. Transitional words should be used. The body paragraph ends with a concluding sentence. Throughout the process, my writing should be coherent and fluent.

4. What is your current knowledge about the formation of a conclusion of a persuasive essay?

It is divided into three minor parts: a restatement of the thesis statement, which is the actual thesis statement with different wording. Then come some suggestions and the clincher.

Participant 3 (S4)

Interview: Phase 1

1. What was your prior knowledge about the structure of a persuasive essay?

I did not have any prior knowledge about the structure of a persuasive essay.

2. What was your prior knowledge about the formation of an introduction of a persuasive essay?

I did not have any idea about the formation of an introduction of a persuasive essay.

3. What was your prior knowledge about the formation of body paragraphs of a persuasive essay?

I did not have any idea about the formation of body paragraphs of a persuasive essay.

4. What was your prior knowledge about the formation of a conclusion of a persuasive essay?

I did not know anything about how to write the conclusion for a persuasive essay, as I did not write it earlier.

Interview: Phase 2

1. What is your current knowledge about the structure of a persuasive essay?

I not only know the structure of a persuasive essay: Introduction, Body paragraphs, and Conclusion, but I am also aware of what to write in different sections of the essay.

2. What is your current knowledge about the formation of an introduction of a persuasive essay?

The idea of adding a grabber, connecting sentences, and the thesis statement is my newfound knowledge. Specially, the thesis statement that contains the entire idea of the essay is something new to me.

3. What is your current knowledge about the formation of body paragraphs of a persuasive essay?

Starting with a topic sentence by taking an idea that is introduced in the thesis statement…continuing with relevant supporting details and ending with a concluding sentence. Transitional words are also necessary to show the shift from one idea to another.

4. What is your current knowledge about the formation of a conclusion of a persuasive essay?

Restatement of the thesis statement, suggestions and the clincher are the elements that I learned to incorporate into the conclusion.

Participant 4 (S4)

Interview: Phase 1

1. What was your prior knowledge about the structure of a persuasive essay?

I wrote argumentative essays at the O level and learned the pattern of an academic essay, but I did not learn persuasive essay writing specifically. Hence, I did not have any knowledge of its exact structure, but in argumentative essays, I followed a structure: Introduction, Body paragraphs, and Conclusion. I am not sure if the same is applicable to persuasive essays.

2. What was your prior knowledge about the formation of an introduction of a persuasive essay?

I was taught to write an interesting introduction that can grab the reader's attention, but it was not for a persuasive essay. In general, I learned to write an introduction like this.

3. What was your prior knowledge about the formation of body paragraphs of a persuasive essay?

I used to write descriptions about the topic in the body paragraph. I used to give suggestions in the body paragraphs. This was my knowledge regarding the components of body paragraphs. I don't know if they correspond to a persuasive essay.

4. What was your prior knowledge about the formation of a conclusion of a persuasive essay?

I used to write the conclusion, but I did not know how to write it. I summarized the information from the body paragraphs in the conclusion.

Interview: Phase 2

1. What is your current knowledge about the structure of a persuasive essay?

Introduction, Body paragraphs and conclusion shape the persuasive essay.

2. What is your current knowledge about the formation of an introduction of a persuasive essay?

Including a grabber, connecting ideas, and a thesis statement is my new learning. Now, I can develop an introduction properly to write, and it is a meaningful learning experience for me.

3. What is your current knowledge about the formation of body paragraphs of a persuasive essay?

Now, I know the proper shape of body paragraphs. Topic sentence at the beginning. Then, I should write some suggestions. Then I have to add the clincher.

4. What is your current knowledge about the formation of a conclusion of a persuasive essay?

The inclusion of a clincher is new to me. In conclusion, I used to write the concluding sentence and add new information, but did not know that the conclusion must be connected with the introduction. Meaning to say, I have to restate the thesis statement and add some suggestions and a clincher.

Participant 5 (S5)

Interview: Phase 1

1. What was your prior knowledge about the structure of a persuasive essay?

I did not have any knowledge about the structure of a persuasive essay, but I wrote descriptive and narrative essays at my O Level. So, I have a prior idea about academic essays. I knew about the structure of an essay.

2. What was your prior knowledge about the formation of an introduction of a persuasive essay?

What to write in the introduction of a persuasive essay was unknown to me, but I used to write some sentences to introduce the topic with some basic information in the introduction of the essays I used to write. I did not write a persuasive essay earlier.

3. What was your prior knowledge about the formation of body paragraphs of a persuasive essay?

I used to form the body paragraphs in my way. I did not know what to write or how to write exactly in response to the topic.

4. What was your prior knowledge about the formation of a conclusion of a persuasive essay?

In conclusion, I used to summarize what I wrote in the introduction and body paragraphs.

Interview: Phase 2

1. What is your current knowledge about the structure of a persuasive essay?

There are three parts of a persuasive essay: Introduction, Body Paragraphs and Conclusion.

2. What is your current knowledge about the formation of an introduction of a persuasive essay?

I have learned to add a grabber, continue with connecting sentences and end with the thesis statement.

3. What is your current knowledge about the formation of body paragraphs of a persuasive essay?

Now, I know the formation of body paragraphs. It should be started with a topic sentence. After that, I have to add supporting ideas/details and end with a concluding sentence.

4. What is your current knowledge about the formation of a conclusion of a persuasive essay?

The inclusion of the thesis statement at the beginning, suggestions in the middle and a clincher at the end form the conclusion.

Participant 6 (S6)

Interview: Phase 1

1. What was your prior knowledge about the structure of a persuasive essay?

I did not have any specific knowledge about the structure of a persuasive essay. In general, I knew that an essay comprises an introduction that contains a definition of the topic, body paragraphs that adds the details of the topic and a conclusion that summarizes the entire write-up.

2. What was your prior knowledge about the formation of an introduction of a persuasive essay?

As I said earlier, I didn't know about the introduction of a persuasive essay. What it contains and how to write it. In general, I knew that an introduction contains a definition of the topic.

3. What was your prior knowledge about the formation of body paragraphs of a persuasive essay?

About the components of the body paragraphs of the persuasive essay, I didn't have specific knowledge.

4. What was your prior knowledge about the formation of a conclusion of a persuasive essay?

I didn't know if the conclusion of a persuasive essay is different from other essays. In an essay, what I knew was a conclusion that summarizes the entire write-up.

Interview: Phase 2

1. What is your current knowledge about the structure of a persuasive essay?

Now, I am aware that the introduction, body paragraphs, and conclusion form a persuasive essay.

2. What is your current knowledge about the formation of an introduction of a persuasive essay?

Adding a grabber is a new learning. Besides, the idea of placing connecting ideas in the middle and the thesis statement at the end enables me to shape my introduction.

3. What is your current knowledge about the formation of body paragraphs of a persuasive essay?

I learn to start with a topic sentence; then, I have to give reasons, which act as supporting details, and add the concluding sentence to end the paragraph. I also learn to avoid adding new ideas in the concluding sentence.

4. What is your current knowledge about the formation of a conclusion of a persuasive essay?

How to begin the conclusion with the restatement of the thesis statement…adding some suggestions in the middle, and clincher at the end.

Participant 7 (S7)

Interview: Phase 1

1. What was your prior knowledge about the structure of a persuasive essay?

I did write essays before, but not persuasive essays. I did not follow any structure. I was not taught this earlier. I did not have any idea about the structure of a persuasive essay.

2. What was your prior knowledge about the formation of an introduction of a persuasive essay?

I did not have any specific knowledge about the elements of an introduction of a persuasive essay.

3. What was your prior knowledge about the formation of body paragraphs of a persuasive essay?

I did not have any specific knowledge about how to form body paragraphs of a persuasive essay.

4. What was your prior knowledge about the formation of a conclusion of a persuasive essay?

I did not have any specific knowledge about the elements of a conclusion of a persuasive essay.

Interview: Phase 2

1. What is your current knowledge about the structure of a persuasive essay?

Introduction, Body paragraphs and conclusion.

2. What is your current knowledge about the formation of an introduction of a persuasive essay?

Now, I am equipped with the knowledge to write an introduction. Grabber, connecting sentences, and the thesis statement are the components of an introduction.

3. What is your current knowledge about the formation of body paragraphs of a persuasive essay?

Starting with a topic sentence by taking an idea from the thesis statement, adding relevant supporting details following transitional words and ending with a concluding sentence…that is what I newly learned.

4. What is your current knowledge about the formation of a conclusion of a persuasive essay?

My current knowledge of constructing a conclusion: stating the restatement of the thesis statement, writing suggestions, and adding a clincher.

Participant 8 (S8)

Interview: Phase 1

1. What was your prior knowledge about the structure of a persuasive essay?

I was unaware of the structure of a persuasive essay, let alone the structure of a persuasive essay.

2. What was your prior knowledge about the formation of an introduction of a persuasive essay?

I had limited knowledge about the components to be included in the introduction of a persuasive essay.

3. What was your prior knowledge about the formation of body paragraphs of a persuasive essay?

I had ideas about writing body paragraphs of an essay, but nothing specific about the ones for a persuasive essay.

4. What was your prior knowledge about the formation of a conclusion of a persuasive essay?

I did not know what to include in the conclusion of a persuasive essay.

Interview: Phase 2

1. What is your current knowledge about the structure of a persuasive essay?

Currently, I know I have to write multiple paragraphs that would represent an introduction, body paragraphs and conclusion.

2. What is your current knowledge about the formation of an introduction of a persuasive essay?

I should include a grabber, connecting sentences, and the thesis statement. These form a proper introduction of a persuasive essay.

3. What is your current knowledge about the formation of body paragraphs of a persuasive essay?

I got the knowledge to add a topic sentence, supporting ideas, and a concluding sentence in the body paragraph.

4. What is your current knowledge about the formation of a conclusion of a persuasive essay?

What I learned after this course is the formation of a conclusion. Now, I know that restatement of the thesis statement should be in there [in the conclusion] at the beginning. It should be followed by some suggestions. Finally, I have to write a clincher.

Participant 1 (S1)

Responses to the open-ended Questionnaire

1. How do you see the contribution of collaborative writing and peer feedback to learning persuasive essay writing?

Engaging in collaborative writing was helpful. We had a discussion before writing the outline. I suggested some irrelevant points... but the other group members corrected me. Feedback from peers improved the quality of the outline of our persuasive essay, as our outline had some flaws. The way we wrote the reasons in the outline did not match the body paragraph's sequence. Peer feedback helped us identify those mistakes, and we got a chance to correct them before the final submission. Moreover, peer feedback helped us write a relevant clincher in their conclusion.

2. Could you share your overall experience of instructional design and materials presented in 'Arwa', collaborative writing and peer feedback concerning your knowledge acquisition subject to persuasive essay writing?

The lecture videos and handouts were useful and easily accessible. I used to watch and read these. Moreover, group work was much needed during COVID-19. It helped a lot. Besides, peer feedback was effective and helpful as it helped us improve our essay writing.

3. Is there anything that you would like to add?

No.

Participant 2 (S2)

Responses to the open-ended Questionnaire

1. How do you see the contribution of collaborative writing and peer feedback to learning persuasive essay writing?

We made a few mistakes in our body paragraphs. Our peers from another group gave us specific advice on where we needed to work on the body paragraphs. We discussed the comments, and we worked collaboratively to correct the mistakes. It's like two-step authentication. First, an individual's understanding was tested through group discussion in collaborative writing. Second, peer feedback further authenticated the correctness of the outline and helped us understand if my group was on the right track or not in the process of writing a persuasive essay.

2. Could you share your overall experience of instructional design and materials presented in 'Arwa', collaborative writing and peer feedback concerning your knowledge acquisition subject to persuasive essay writing?

Lecture videos and handouts were helpful because I could learn independently. During the COVID-19 days, I lost motivation. My group members motivated me to learn the content and get involved in the writing process. Eventually, I watched the videos and read the handouts…Peer feedback allowed us to see concrete examples of what worked and what needed to be improved in our writing.

3. Is there anything that you would like to add?

No.

Participant 3 (S3)

Responses to the open-ended Questionnaire

1. How do you see the contribution of collaborative writing and peer feedback to learning persuasive essay writing?

Working in groups helped address the knowledge gap. Peer feedback helped us write relevant supporting details that are related to the topic sentence. Due to peer feedback, we managed to write the restatement of the thesis statement properly and add relevant suggestions.

2. Could you share your overall experience of instructional design and materials presented in 'Arwa', collaborative writing and peer feedback concerning your knowledge acquisition subject to persuasive essay writing?

Independent learning was made easier with the help of lecture videos and handouts. Despite a period of tough times due to my uncle's death, my group members encouraged me. They guided me to learn from the online resources. Eventually, I was able to catch up on the videos and readings. The deconstructed version of the essay in the handout was specially effective.

3. Is there anything that you would like to add?

No.

Participant 4 (S4)

Responses to the open-ended Questionnaire

1. How do you see the contribution of collaborative writing and peer feedback to learning persuasive essay writing?

Collaborative writing and peer feedback fostered my learning of persuasive essay writing.

2. Could you share your overall experience of instructional design and materials presented in 'Arwa', collaborative writing and peer feedback concerning your knowledge acquisition subject to persuasive essay writing?

I could get access to the lecture videos and handouts at my convenience. It was helpful. The knowledge gap among the group members was bridged during the collaborative writing process. Moreover, peer feedback generated further guidance.

3. Is there anything that you would like to add?

No.

Participant 5 (S5)

Responses to the open-ended Questionnaire

1. How do you see the contribution of collaborative writing and peer feedback to learning persuasive essay writing?

Collaborative writing and peer feedback helped…Through peer feedback, we learned that topic sentences were not relevantly aligned with the thesis statement. Again, we collaboratively dealt with the comment and revised it to include a relevant topic sentence in alignment with the thesis statement.

2. Could you share your overall experience of instructional design and materials presented in 'Arwa', collaborative writing and peer feedback concerning your knowledge acquisition subject to persuasive essay writing?

Undoubtedly, I benefited from the instructional design and materials presented in 'Arwa'. Collaborative writing and peer feedback were useful, too.

3. Is there anything that you would like to add?

No.

Participant 6 (S6)

Responses to the open-ended Questionnaire

1. How do you see the contribution of collaborative writing and peer feedback to learning persuasive essay writing?

The collaborative writing and peer feedback significantly contributed to my learning.

2. Could you share your overall experience of instructional design and materials presented in 'Arwa', collaborative writing and peer feedback concerning your knowledge acquisition subject to persuasive essay writing?

The availability of the materials and resources, along with online sessions with teachers, helped me to learn how to write a persuasive essay. The collaborative writing and peer feedback were also helpful.

3. Is there anything that you would like to add?

No.

Participant 7 (S7)

Responses to the open-ended Questionnaire

1. How do you see the contribution of collaborative writing and peer feedback to learning persuasive essay writing?

Collaborative writing and peer feedback were conducive. While my group completed the first draft of the essay, another group confirmed its correctness. They focused on what we 'did well' in the essay. This time, they did not elicit anything under the 'could improve', which was an indication of our understanding of persuasive essay writing.

2. Could you share your overall experience of instructional design and materials presented in 'Arwa', collaborative writing and peer feedback concerning your knowledge acquisition subject to persuasive essay writing?

Alongside lecture videos and handouts, peer feedback was also a crucial part of our success, as it gave us concrete examples of what worked and what needed to be improved in our writing.

3. Is there anything that you would like to add?

No.

Participant 8 (S8)

Responses to the open-ended Questionnaire

1. How do you see the contribution of collaborative writing and peer feedback to learning persuasive essay writing?

Peer feedback helped us include all the components of the introduction, maintaining a proper order. Together, we made the mistake. Together, we learned to correct it. So, collaborative writing and peer feedback were supportive to us.

2. Could you share your overall experience of instructional design and materials presented in 'Arwa', collaborative writing and peer feedback concerning your knowledge acquisition subject to persuasive essay writing?

I received great support because of the instructional design and materials presented in 'Arwa'. Collaborative writing and peer feedback were specially helpful.

3. Is there anything that you would like to add?

No.
